# The differences in drug resistance between drug-resistant tuberculosis patients with and without diabetes mellitus in northeast China: a retrospective study

**DOI:** 10.1186/s12879-023-08130-1

**Published:** 2023-03-15

**Authors:** Yuanping Pan, Yingying Yu, Yaohui Yi, Xiaofeng Dou, Jiachen Lu, Ling Zhou

**Affiliations:** School of Public Health, Dalian Medical University, 9 West Section, Lushun South Road, Dalian, Liaoning Province People’s Republic of China

**Keywords:** Drug-resistant tuberculosis, Diabetes mellitus, Resistance profile, Trends, Risk factors

## Abstract

**Background:**

Diabetes mellitus (DM) and drug-resistant tuberculosis (DR-TB) are serious global public health problems. This study aimed to explore the differences in drug resistance between DR-TB patients with and without DM. Risk factors for developing multidrug-resistant tuberculosis (MDR-TB) were also investigated among DR-TB patients.

**Methods:**

The patient’s basic demographic,　clinical characteristics, and drug susceptibility testing (DST) data were collected from the Chinese Disease Control Information System. Descriptive statistics were used to estimate the frequency and proportion of included variables. Categorical variables were compared using the Chi-square test or Fisher’s exact test. Chi-square tests for trends were used to determine changes and trends in MDR-TB and pre-extensively drug-resistantTB (pre-XDR-TB) patterns over time. Univariate and multivariate logistic regression analysis was used to explore the risk factors of MDR-TB.

**Results:**

Compared with DR-TB patients with DM, DR-TB patients without DM had significantly higher rates of mono-resistant streptomycin (SM) and any resistance to kanamycin (KM), but significantly lower rates of any resistance to protionamide (PTO) and mono-resistance to levofloxacin (LFX), and pre-XDR-TB (*P*<0.05). The proportion of resistance to other anti-TB drugs was not statistically different between the DR-TB with and without DM. Among DR-TB patients without and with DM, the proportion of patients with MDR-TB and pre-XDR-TB patterns showed a significant downward trend from 2016 to 2021 (*P*<0.05). Among DR-TB patients without DM, male, previously treated DR-TB cases, and immigration were risk factors for MDR-TB (*P*<0.05). In DR-TB patients with DM, a negative sputum smear is a risk factor for MDR-TB (*P*<0.05).

**Conclusion:**

There was no statistical difference in resistance patterns between DR-TB with and without DM, except in arbitrary resistance to PTO and KM, mono-resistant SM and LFX, and pre-XDR-TB. Great progress has been made in the prevention and control of MDR-TB and pre-XDR-TB. However, DR-TB patients with and without DM differ in their risk factors for developing MDR-TB.

## Background

Tuberculosis (TB) is an infectious disease caused by *Mycobacterium tuberculosis* which seriously damages human health, especially in some low- and middle-income population. In 2021, 10.6 million new cases of TB were diagnosed globally, with an incidence rate of 134 per 100,000 and 1.6 million deaths from TB[1]. The World Health Organization (WHO) proposed a strategy to eliminate TB, which was to reduce TB mortality to less than 95% and incidence to 90% by 2035[2]. However, the emergence of drug-resistant TB (DR-TB) has seriously hindered the prevention and treatment of TB[3]. DR-TB is now becoming the world’s deadliest pathogen, with a quarter of deaths attributed to antimicrobial drug resistance[4]. There are nearly 5000,000 patients with rifampicin-resistance TB (RR-TB) worldwide, of which 78% were multidrug-resistant TB (MDR-TB), defined as resistance to at least isoniazid (INH) and rifampicin (RFP), reported in 2019[5]. Resistance to anti-TB drugs often means fewer treatments, poorer outcomes, and higher medical costs. Knowledge of drug resistance profiles can help us evaluate current TB control measures and develop more effective TB treatments.

In countries with a high TB burden, an estimated 15% of TB patients have diabetes mellitus (DM)[6]. With changing lifestyles and aging population, International Diabetes Federation (IDF) estimated that around 537 million adults worldwide will have DM in 2021, and that number is expected to rise to 643 million by 2030[7]. Due to the high prevalence of TB and DM, the double burden of TB and DM constitutes a global public health concern[8]. China has one of the highest TB burdens in the world, and previous studies have shown a significant burden of DR-TB in northeastern China[9, 10]. Moreover, northeastern China has a significant aging population and a high burden of DM[11]. The relationship between DM and TB has been investigated, and previous studies identified it as a risk factor for developing TB[12–14]. In addition, the impact of DM on TB treatment outcomes has been extensively studied and there is broad consensus that the relationship between DM and TB treatment outcomes.[15–17]. However, few studies have looked at the relationship between DR-TB and DM. A study was conducted to explore the profiles of TB patients with different DM statuses in eastern China, and it did not find an association between DM and DR-TB[18]. Conversely, other studies have found that DM is a risk factor for DR-TB in TB patients[8, 19]. Due to DM complicating the treatment of DR-TB, the research on this aspect is relatively insufficient. This study focused on the differences in drug resistance between DR-TB patients with and without DM, and finally explored the risk factors for developing MDR-TB among DR-TB patients with and without DM.

## Methods

### Study population and data collection

**Fig. 1:**
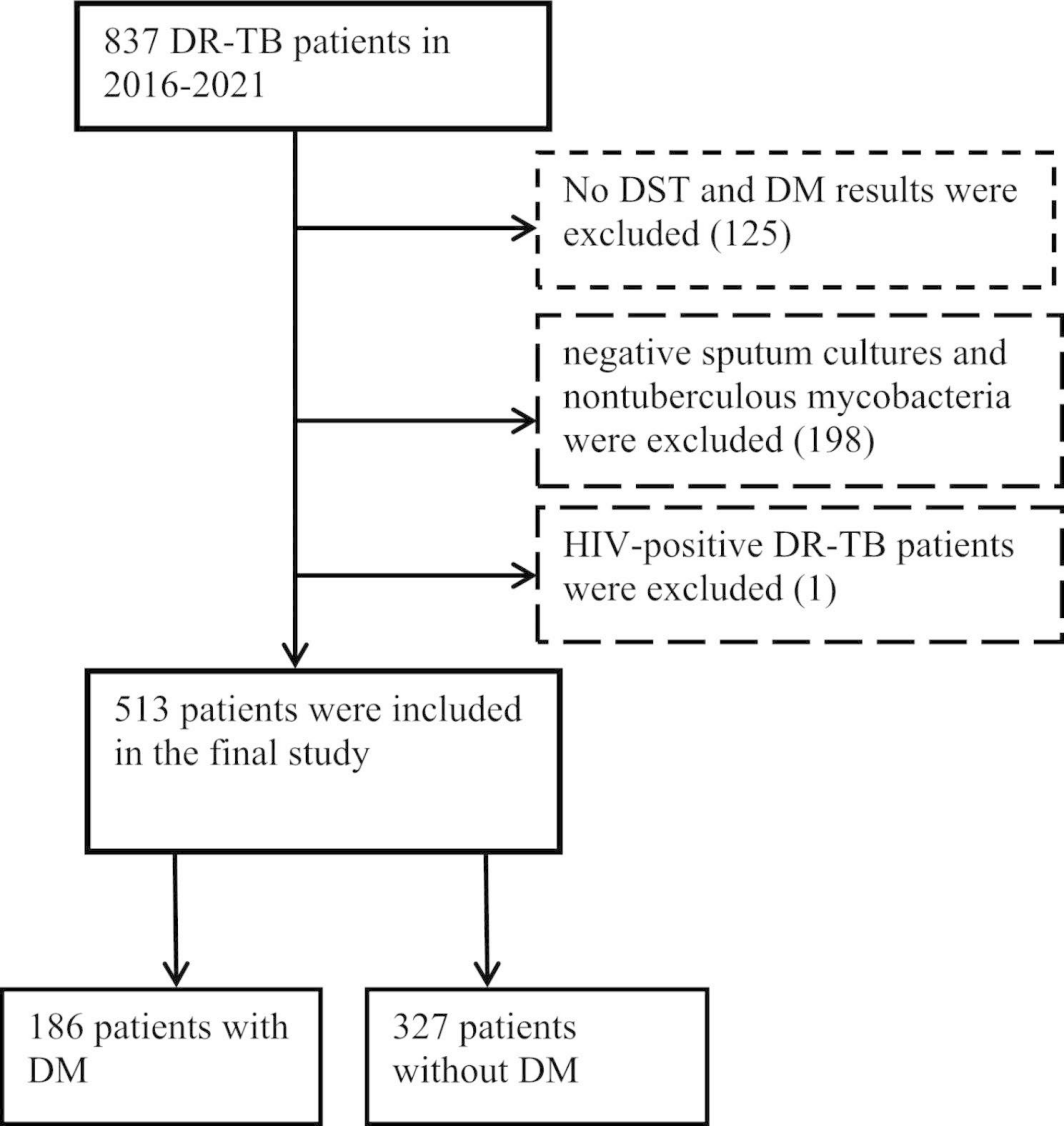
Flowchart for the inclusion of patients Abbreviations: DR-TB, drug-resistant tuberculosis; DM, diabetes mellitus; DST, drug susceptibility testing.

This was a retrospective study conducted at a specialized TB hospital in northeastern China. All sociodemographic, clinical, and drug susceptibility data from 1 to 2016 to 31 December 2021 were extracted from the China Disease Control and Prevention Information System which regularly collects information associated with DR-TB surveillance and management. The sociodemographic included sex (female/male), age (14~ 30/31 ~ 44/45 ~ 59/60 aged and above), residence (urban/rural), nationality (Han/others), occupation (employed/unemployed), migrant (yes/no). Clinical data included patient category (primary DR-TB cases / previously treated DR-TB cases), sputum smear status (negative/active), and DM status. Drug susceptibility testing (DST) data included the testing results of first-line drugs and second-line drugs. Patients with negative sputum cultures and nontuberculous mycobacteria were excluded. Patients without DST results were excluded. Since HIV-positive patients are treated in specialized sentinel hospitals, data related to treatment are not available. Therefore, we excluded HIV-positive DR-TB patients. In addition, patients without results of DM also were excluded. A total of 513 patients were included in the final study, including 186 patients with DM and 327 patients without DM (Fig. 1).

### Drug Sensitivity Testing

Sputum samples were cultured on Lowenstein-Jensen (LJ) culture media[20]. DST was conducted for first-line anti-TB drugs including INH, RFP, ethambutol (EMB), and streptomycin (SM), and for second-line drugs including protionamide (PTO), para-aminosalicylic acid (PAS), amikacin (AM), capreomycin (CM), kanamycin (KM), levofloxacin (LFX) and ofloxacin (OFX). The following concentrations of anti-TB drugs were administered according to the proportion method: 0.2 µg/ml for INH, 40.0 µg/ml for RIF, 2.0 µg/ml for EMB, 4.0 µg/ml for SM, 30.0 µg/ml for KM, 2.0 µg/ml for OFX, 2.0 µg/ml for LFX, 40.0 µg/ml for CM, 40.0 µg/ml for AM, 1.0 µg/ml for PAS and 40.0 µg/ml for PTO[21]. Resistance to a specific drug was determined if the growth rate was greater than 1.0% compared to the control[21].

### Definitions

According to the report issued by WHO, relevant definition of TB defined[22].

- Pre-XDR-TB was defined as being resistant to at least INH and RFP, as well as to either of the three injectable second-line drugs or to ofloxacin.
- XDR-TB was defined as resistance to at least INH and RFP, combined with resistance to a fluoroquinolone and resistance to one of three injectable second-line drugs.
- Primary DR-TB cases refer to a patient who has never been treated for TB or has taken TB treatment for less than one month.
- Previously treated DR-TB cases referred to patients who had received one month or more of TB treatment in the past.
- Mono-resistant TB (MR-TB) is defined as resistance to one first-line anti-TB drug only.
- Polydrug-resistant TB (PDR-TB) is defined as resistance to more than one first-line anti-TB drug (other than both INH and RFP).
- Patients with DM were defined as those with fasting plasma glucose ≥ 126 mg/dL (7 mmol/L) or hemoglobin A1C ≥ 6.5% or who self-reported having been diagnosed with diabetes by a physician[23].

### Statistical analysis

The demographics, clinical, and the results of DST for DR-TB patients with and without DM were depicted using descriptive statistics. The categorical characteristics of DR-TB patients were compared by DM status using Chi-square test or Fisher’s exact. In addition, Chi-square tests for trends were used to determine changes and trends in MDR-TB and pre-XDR-TB patterns over time. To identify the link between a collection of independent variables and dependent variables, multivariate logistic regression was performed on all variables with *P*-values < 0.25 in univariate logistic regression. Two-sided *P* < 0.05 was considered statistically significant. All data analysis was carried out by using SPSS 20.0 statistical package (IBM Corporation, Armonk, State of New York).

## Results

### Patients’ characteristics

**Figure 2:**
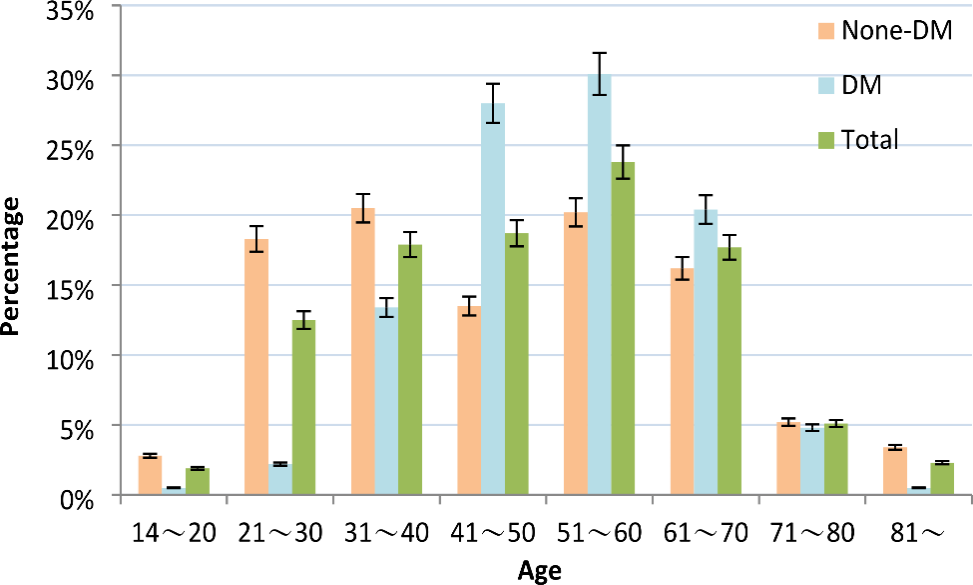
Proportion of DR-TB with and without DM patients in different age groups Abbreviations: DM, diabetes mellitus.

A total of 513 DR-TB patients were included in the present study. Of these patients, 327 (63.74%) patients did not have DM; 186 (36.26%) patients had DM. The percentage of the male was 62.08% and 86.02% among DR-TB patients without and with DM, respectively (*P*<0.05). Of all patients, 34.11% were between 45 and 59 years of age and 27.49% were over 60 years of age. In addition, In terms of overall patients, the highest percentage of patients were 51~60 years old. The highest percentage of non-DM patients with DR-TB was in the age group of 31~40 years, but the highest percentage of DM patients with DR-TB was in the age group of 51~60 years (Fig. 2). Among DR-TB patients without and with DM, the percentage of urban residents was 87.77% and 89.25%, respectively. Patients with a history of previous tuberculosis treatment were 64.22% and 60.75% among DR-TB patients without and with DM, respectively. The majority of patients had positive sputum smears at baseline (81.35% vs. 85.48% ). Unemployment was 88.07% among DR-TB patients without diabetes and 89.78% among DR-TB patients with diabetes. The vast majority of patients are Han Chinese (98.47% vs. 97.85%). The percentage of migrants was 39.76% and 36.02% among DR-TB patients without and with DM, respectively (**Table ****1**).

**Table 1:** Demographic and clinical characteristics of DR-TB patients without and with DM.

Characteristics	No DM, N (%)	DM, N (%)	Total, N (%)	χ^2^	*P-*value
Gender				32.847	<0.001
Male	203 (62.08)	160 (86.02)	363 (70.76)		
Female	124 (37.92)	26 (13.98)	150 (29.24)		
Age (years)				52.078	<0.001
14~ 30	69 (21.10)	5 (2.69)	74 (14.42)		
31 ~ 44	87 (26.61)	36 (19.35)	123 (23.98)		
45 ~ 59	82 (25.08)	93 (50.00)	175 (34.11)		
60~	89 (27.22)	52 (27.96)	141 (27.49)		
Patient residence				0.251	0.616
Urban	287 (87.77)	166 (89.25)	453 (88.30)		
Rural	40 (12.23)	20 (10.75)	60 (11.70)		
Patient Category				0.611	0.434
Primary DR-TB cases	117 (35.78)	73 (39.25)	190 (37.04)		
Previously treated DR-TB cases	210 (64.22)	113 (60.75)	323 (62.96)		
Sputum smear status				1.429	0.232
Negative	61 (18.65)	27 (14.52)	88 (17.15)		
Active	266 (81.35)	159 (85.48)	425 (82.85)		
Occupation				0.346	0.556
Unemployed	288 (88.07)	167 (89.78)	455 (88.69)		
Employed	39 (11.93)	19 (10.22)	58 (11.31)		
Nationality					
Others	5 (1.53)	4 (2.15 )	9 (1.75)	0.197	0.657
Han	322 (98.47)	182 (97.85)	504 (98.25)		
Migrant				0.699	0.403
No	197 (60.24)	119 (63.98)	316 (61.60)		
Yes	130 (39.76)	67 (36.02)	197 (38.40)		

Abbreviations: DM, diabetes mellitus; DR-TB, drug-resistant tuberculosis.

Notes: Migrant refers to people who leave their domicile for various reasons and go to other Provinces and cities with registered tuberculosis patients.

### Drug resistance profile

As is shown in Table 2, among 327 patients without DM and 186 patients with DM, 44,95% and 47.85% were any INH resistant, respectively; 97.55% and 99.46% were any RFP resistant respectively. The ratio of mono-resistance to SM in cases with and without DM were 6.73% and 2.69%, respectively. As is shown in Table 3, 1.83% and 5.38% were any PTO resistant, respectively among 327 patients without DM and 186 patients with DM. 7.65% and 2.69% were any KM resistant, respectively. The ratio of mono-resistance to LFX among cases with and without DM were 2.75% and 6.45%, respectively. Among 327 cases without DM and 186 cases with DM, 7.34% and 13.98% were pre-XDR-TB, respectively

**Table 2:** First-line drugs resistance profile DR-TB patients without diabetes mellitus and with diabetes mellitus

Drug resistance pattern	No DM, N (%)	DM, N (%)
Any resistance		
Any INH	147 (44.95)	89 (47.85)
Any RFP	319 (97.55)	185 (99.46)
Any EMB	53 (16.21)	31 (16.67)
Any SM	102 (31.19)	59 (31.72)
MR-TB		
INH	5 (1.53)	1 (0.54)
RFP	159 (48.62)	91 (48.92)
EMB	0 (0)	0 (0)
SM	22 (6.73)	5 (2.69)
PDR-TB		
RFP + SM	17 (5.20)	5 (2.69)
INH + SM	12 (3.67)	6 (3.23)
INH + EMB	0 (0)	1 (0.54)
RFP + EMB + SM	3 (0.92)	0 (0)
MDR-TB		
INH + RFP	130 (39.76)	82 (44.09)
INH + RFP + EMB	46 (14.07)	29 (15.59)
INH + RFP + SM	42 (12.84)	25 (13.44)
INH + RFP + EMB + SM	27 (8.26)	22 (11.83)

Abbreviations: INH, isoniazid; RFP, rifampicin; SM, streptomycin; EMB, ethambutol; MR-TB, mono-resistant tuberculosis; PDR-TB, polydrug-resistant tuberculosis; MDR-TB, multidrug-resistant tuberculosis; DM, diabetes mellitus.

**Table 3:** Second-line drugs resistance profile DR-TB patients without and with DM.

Drugs resistance pattern	No DM, N (%)	DM, N (%)
Any resistance to second-line drugs		
Any PTO	6 (1.83)	10 (5.38)
Any PAS	19 (5.81)	8 (4.30)
Any AM	23 (7.03)	6 (3.23)
Any CM	13 (3.98)	9 (4.84)
Any KM	25 (7.65)	5 (2.69)
Any LFX	50 (15.29)	35 (18.82)
Any OFX	51 (15.60)	25 (13.44)
Monoresistant second-line drugs		
PTO	1 (0.31)	3 (1.61)
PAS	4 (1.22)	4 (2.15)
AM	1 (0.31)	0 (0)
CM	1 (0.31)	3 (1.61)
KM	2 (0.61)	2 (1.08)
LFX	9 (2.75)	12 (6.45)
OFX	10 (3.06)	7 (3.76)
Pre-XDR-TB	24 (7.34)	26 (13.98)
XDR-TB	16 (4.89)	5 (2.69)

Abbreviations: PTO, protionamide; PAS, para-aminosalicylic acid; AM, amikacin; CM, capreomycin; KM, kanamycin; LFX, levofloxacin; OFX, ofloxacin; XDR-TB, extensively drug-resistant TB; DM, diabetes mellitus.

### Annual drug resistance trends for MDR and pre-XDR-TB from 2016 to 2021

**Figure 3:**
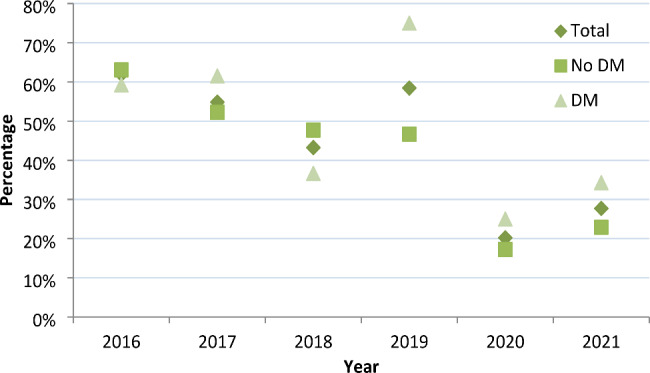
Trends in the proportion of MDR among DR-TB patients without and with DM from 2016 to 2021 Abbreviations: DM, diabetes mellitus.

**Figure 4:**
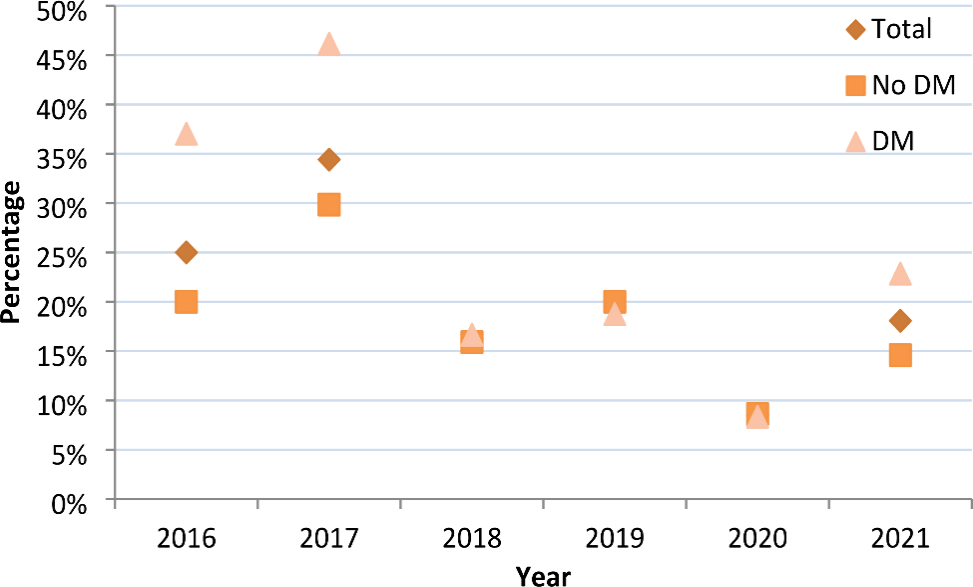
Trends in the proportion of pre-XDR-TB among DR-TB patients without and with DM from 2016 − 2021 Abbreviations: DM, diabetes mellitus.

Among the 513 patients, the rate of MDR-TB decreased from 61.96% in 2016 to 27.71% in 2021, with an average annual decrease of 14.86% (*P*<0.05). The rate of pre-XDR-TB decreased from 25.00% in 2016 to 18.07% in 2021, with an average annual decrease of 6.28% (*P*<0.05). Among 327 patients without DM, the rate of MDR-TB decreased from 63.08% in 2016 to 22.92% in 2021, with an average annual decrease of 18.33% (*P*<0.05). The rate of pre-XDR-TB decreased from 20.00% in 2016 to 14.58% in 2021, with an average annual decrease of 6.12% (*P*<0.05). Among 186 patients with DM, the rate of MDR-TB decreased from 59.26% in 2016 to 34.29% in 2021, with an average annual decrease of 10.37% (*P*<0.05). The rate of pre-XDR-TB decreased from 37.04% in 2016 to 22.86% in 2021, with an average annual decrease of 9.20% (*P*<0.05)(**Fig. ****3** and **Fig. ****4**)

### Risk of MDR-TB in patients with DR-TB combined without and with DM

In the multivariable analysis, among patients without DM, compared with women, men had a 1.71-fold risk of developing MDR-TB (OR = 1.71, 95% CI: 1.04–2.82, *P*<0.05); previously treated DR-TB cases had a 1.70-fold risk of developing MDR-TB compared with primary DR-TB cases (OR = 1.70, 95% CI: 1.04–2.79, *P*<0.05). Immigrants are 2.08 times more likely to develop MDR-TB than native residents (OR = 2.08, 95% CI: 1.28–3.39, *P*<0.05) (**Table ****4**). However, among patients with DM, negative sputum smear alone was an independent risk factor for developing MDR-TB (OR = 2.65, 95% CI: 1.04–6.76, *P*<0.05) (**Table ****5**).

**Table 4:** Risk factors of MDR-TB among DR-TB patients without DM.

Characteristics	None-MDR N(%)	MDR N(%)	OR(95%CI)	*P*-value	AOR(95%CI)	*P*-value
Gender						
Female	66 (35.11)	58 (41.73)	1		1	
Male	122 (64.89)	81 (58.27)	0.76 (0.48–1.19)	0.223	1.71 (1.04–2.82)	0.034
Age (years)						
14~ 30	39 (20.74)	30 (21.58)	1		1	
31–44	60 (31.91)	27 (19.42)	0.59 (0.30–1.13)	0.110	0.56 (0.28–1.11)	0.098
45–59	42 (22.34)	40 (28.78)	1.24 (0.65–2.36)	0.515	1.45 (0.73–2.91)	0.290
60	47 (25.00)	42 (30.22)	1.16 (0.62–2.19)	0.642	1.42 (0.71–2.83)	0.319
Patient residence						
Rural	24 (12.77)	16 (11.51)	1		-	-
Urban	164 (87.23)	123 (88.49 )	1.13 (0.57–2.21)	0.732	-	
Patient Category						
Primary DR-TB cases	76 (40.43)	41(29.50)	1		1	
Previously treated DR-TB cases	112 (59.57)	98 (70.50)	1.62 (1.02–2.59)	0.042	1.70 (1.04–2.79)	0.033
Occupation						
Unemployed	162 (86.17)	126 (90.65)	1		1	
Employed	26 (13.83)	13 (9.35)	0.63 (0.31–1.30)	0.220	0.78 (0.36–1.66)	0.512
Nationality						
Han	184 (97.87)	138 (99.28)	1		-	-
Others	4 (2.13)	1 (0.72)	3.00 (0.33–27.14)	0.328	-	
Migrant						
No	127 (67.55)	70 (50.36)	1		1	
Yes	61 (32.45)	69 (49.64)	2.05(1.31–3.22)	0.002	2.08 (1.28–3.39)	0.003
Sputum smear status						
Active	161 (85.64)	105 (75.54)	1		1	
Negative	27 (14.36)	34 (24.46)	1.93 (1.0-3.39)	0.022	1.72 (0.94–3.17)	0.079

Abbreviations: CI, Confidence Interval; OR, Odds Ratio; AOR, Adjust Odds Ratio, MDR-TB, multi-drug resistant tuberculosis.

Notes: Migrant refers to people who leave their domicile for various reasons and go to other Provinces and cities with registered tuberculosis patients.

**Table 5:** Risk factors of MDR-TB among DR-TB patients with DM.

Characteristics	None-MDR, N (%)	MDR, N (%)	OR (95%CI)	*P*-value	AOR (95%CI)	*P*-value
Gender						
Female	14 (14.29)	12 (13.64)	1		-	-
Male	84 (85.71)	76 (86.36)	1.06 (0.46–2.42)	0.899	-	
Age (years)						
14~ 30	2 (2.04)	3 (3.41)	1		-	-
31–44	18 (18.37)	18 (20.45)	0.67 (0.10–4.48)	0.677	-	
45–59	54 (55.10)	39 (44.32)	0.48 (0.08–3.02)	0.435	-	
60	24 (24.49)	28 (31.82)	0.78 (0.12–5.05)	0.792	-	
Patient residence						
Rural	11 (11.22)	9 (10.23)	1		-	-
Urban	87 (88.78)	79 (89.77)	1.11 (0.44–2.82)	0.827	-	
Patient Category						
Primary DR-TB cases	45 (45.92)	28 (31.82)	1		1	
Previously treated DR-TB cases	53 (54.08)	60 (68.18)	1.82 (1.00-3.31)	0.050	1.85(1.00-3.42)	0.051
Occupation						
Unemployed	86 (87.76)	81 (92.05)	1		-	-
Employed	12 (12.24)	7 (7.95)	0.62 (0.23–1.65)	0.338	-	
Nationality						
Han	97 (98.98)	85 (96.59)	1		-	-
Others	1 (1.03)	3 (3.41)	3.00 (0.33–27.14)	0.328	-	
Migrant						
No	70 (71.43)	49 (55.68)	1		1	
Yes	28 (28.57)	39 (44.32)	2.05 (1.31–3.22)	0.002	1.58 (0.83–3.02)	0.168
Sputum smear status						
Active	90 (8.16)	69 (21.59)	1		1	
Negative	8 (91.84)	19 (78.41)	3.10 (1.28–7.50)	0.012	2.65 (1.04–6.76)	0.042

Abbreviations: CI, Confidence Interval; OR, Odds Ratio; AOR, Adjust Odds Ratio, MDR-TB, multi-drug resistant tuberculosis.

Notes: Migrant refers to people who leave their domicile for various reasons and go to other provinces and cities with registered tuberculosis patients.

## Discussion

This is the first study on the difference in drug resistance profile and trends of DR-TB patients with and without DM in northeast China. In our study, we found that patients with DM were older and were more likely to be men than patients without DM. These findings were similar to studies conducted in Georgia and China[24, 25]. Some reasons can be explained. On the one hand, male and older age are independent risk factors for DM, as many studies have confirmed[26–28]. In addition, men tend to be more likely to develop DR-TB than women[29]. However, some studies have also reported that women are at high risk for DR-TB[30, 31]. This difference may be due to socioeconomic and cultural differences and regional differences. On the other hand, the population of northeast China is seriously aging, and in 2020, the proportion of elderly people over 60 years old would exceed 20%. In this study, patients aged 60 and over accounted for 27.49%, which may be the reason for the larger proportion of elderly patients in patients without and with DM.

Except for mono-resistant SM, the drug resistance profile of other first-line anti-TB drugs in patients with and without diabetes was not statistically different. This result is similar to that of a previous study[18], but studies in other countries have come to the opposite conclusion[8, 19]. We speculate that may be due to different study designs and smaller samples. The difference in mono-resistant SM between the DR-TB with and without DM implies that our health care providers are aware of this difference when dealing with patients with and without DM when administering clinical drug therapy. In total, the proportion of patients with DM was higher for arbitrary PTO resistance, and for mono-resistant of LFX than for patients without diabetes. However, among patients with without DM, arbitrary KM resistance, and mono-resistant of SM were higher than that of patients with DM. In addition to this, we also found that the proportion of pre-XDR-TB was significantly higher in patients with DM compared to those without DM. In other studies, some opposite findings were found[18]. Molecular epidemiology should be further developed to examine the reasons for this discrepancy.

Our study found that MDR-TB and pre-XDR-TB with and without DM showed a downward trend from 2016 to 2021 (*P*<0.05). We speculate that this may be since two-way screening for TB and DM was carried out according to the requirements of the National Health Commission of China, allowing more patients to receive active treatment and preventing the spread of DR-TB. However, it is worth noting the increasing trend of MDR and pre-XDR-TB resistance rates from 2020 to 2021, which may be due to the impact of the COVID-19 pandemic. Among patients without DM, male, a history of TB treatment and migration are independent risk factors for the development of MDR-TB. Males are more likely to smoke and abuse alcohol than women, both of which are risk factors for developing MDR-TB[32]. In addition, men are more likely than women to be infected with drug-resistant *Mycobacterium tuberculosis* because their work involves more social interaction[33]. Patients with a history of TB treatment may have their sensitive *Mycobacterium tuberculosis* eliminated and drug-resistant *Mycobacterium tuberculosis* left behind due to multiple treatments[34]. Immigrants are not often able to regularize their treatment due to their unstable work and place of residence, which eventually leads to the development of drug resistance[35]. Among patients with DM, sputum-smear-negative patients were more likely to develop MDR-TB than sputum-smear-positive patients. This may be since patients with negative sputum smears receive less attention and guidance from medical staff than patients with positive sputum smears, leading to irregular medication and more likely to develop MDR-TB.

There are some limitations. First, relatively few patients were included in our study, especially those with DR-TB and DM. Second, because our data came from the Chinese Disease Control Information System, some important information such as smoking, drinking, and adverse reactions were not found in this system. Third, some important anti-tuberculosis drugs, such as moxifloxacin and pyrazinamide, were not included due to many deletions. Fourth, the exclusion of HIV-positive patients may not explore the underlying status of drug resistance among HIV-positive drug-resistant patients. Nonetheless, our study identified differences in drug resistance patterns and trends among DM and non-DM patients with DR-TB, as well as risk factors for developing MDR-TB. These findings provide important information for healthcare workers to combat DR-TB.

## Conclusion

Except for arbitrary resistance to PTO and KM, mono-resistance to SM and LFX, and pre-XDR-TB, there was no statistical difference in resistance patterns between patients with and without DM. Among patients with and without DM, the highest proportion of drug resistance was RFP. Great progress has been made in the prevention and treatment of DR-TB in patients with and without DM. Male gender, history of TB treatment, and migrant are risk factors for MDR-TB in non-DM patients with DR-TB. However, in patients with DR-TB and DM, negative sputum smear is a geographic risk factor for developing MDR-TB. The development of targeted measures among DR-TB patients with and without DM would be beneficial for the control of DR-TB.

## Data Availability

The datasets analysed in the present study are available from the corresponding author on reasonable request.
